# Living with metastatic breast cancer (LIMBER): experiences, quality of life, gaps in information, care and support of patients in the UK

**DOI:** 10.1007/s00520-023-07928-8

**Published:** 2023-07-11

**Authors:** L. Fallowfield, R. Starkings, C. Palmieri, A. Tait, L. Stephen, S. May, R. Habibi, S. Russ, V. Shilling, V. Jenkins

**Affiliations:** 1 Sussex Health Outcomes Research & Education in Cancer (SHORE-C), Brighton & Sussex Medical School, University of Sussex, Brighton, UK; 2 Department of Molecular and Clinical Cancer Medicine, University of Liverpool, Liverpool, UK; 3 Department of Medical Oncology, The Clatterbridge Cancer Centre NHS Foundation Trust, Birkenhead, UK; 4 Make2ndsCount, Edinburgh, UK

**Keywords:** Advanced/metastatic/secondary breast cancer, Communication, Information needs, Patient online survey, Support resources

## Abstract

**Purpose:**

To determine the experiences, information, support needs and quality of life of women in the UK living with metastatic breast cancer (MBC) to provide content for educational materials.

**Methods:**

An online survey, hosted for 3 months on a UK MBC charity website, comprised sections covering issues such as communication about MBC treatment and management, helpful and less helpful things that healthcare professionals, family and friends did or said and completion of the Patient Roles and Responsibilities Scale (PRRS).

**Results:**

A total of 143 patients participated; 48/143(33%) presented de novo; 54/143(38%) had been living with MBC > 2 years. PRRS analysis revealed that MBC imposed a serious impact upon most respondents’ own caring abilities and social lives. A majority 98/139 (71%) wished they had known more about MBC before their diagnosis; 63/134(47%) indicated that they still did not fully understand their illness; merely 78/139(56%) had access to a specialist nurse and only 69/135(51%) had been offered any additional support. Respondents reported little consideration given to their lifestyle/culture during consultations and inconsistent information, support services, continuity of care or access to clinical trials. They commented upon things health care professionals/friends and family did or said that were useful and cited other behaviours that were especially unhelpful.

**Conclusions:**

MBC exerted a deleterious impact upon patients’ activities of daily living which were exacerbated in part by significant gaps in support, communication and information.

**Implications for cancer survivors:**

LIMBER results are informing the content of educational materials currently being developed for patients’ formal and informal carers.

## Introduction

There are now an estimated 67,000 people living with metastatic breast cancer (MBC) in the UK [1]. Although many therapeutic advances made during the last 5 years have led to significant improvements in the overall survival of patients with MBC, fewer than 3% will live longer than 5 years after their diagnosis [2]. Preserving the quality of that survival time is vital and there are questions as to whether or not patients are provided with sufficient understandable and individually relevant information to make informed decisions about the available treatment options. Although most clinical trials do now include some assessment of quality of life, the patient-reported outcome measures are often rather limited, for example there is an over reliance on toxicity assessment using physician reported CTCAE grading [3]. Few clinical treatment trials, for example, measure the impact that toxicities regarded as ‘manageable’ exert on patients’ abilities to perform important roles and responsibilities. Resources that provide such information together with appropriate support and ameliorative interventions are an essential part of decision-making and the care of patients with MBC.

The experiences of women who have secondary disease differ from those with early breast cancer. The latter have realistic hopes of cure, whilst the former face the prospect of lifelong tests and treatment with considerable uncertainty about their future. In most breast cancer centres, clinical nurse specialists guide, help and support women with early disease. In contrast, those with MBC rarely have access to similar services. It cannot be assumed that all patients will receive appropriate help from their partners, family or friends or that they have sufficient self-efficacy to question the lack of resources.

There are fewer publications regarding the views, perceptions and experiences of women with more advanced disease compared to those in early breast cancer [4, 5]. One study reported that some witnessed fear, disinterest and uncomfortable exchanges with other people [6] whilst another cited the lack of interest displayed in them over time resulting in isolation [7]. Few patients however inhabit social vacuums, so the quality of their survival can be seriously affected by the ability of family members and close friends to deal with the emotional and practical consequences of a loved one with advanced disease.

The broader effects and implications of living with cancer such as financial burdens (financial toxicity) incurred including a loss of income from employment, difficulties in coping with changing family dynamics and how all of this may impact relationships are, generally speaking, under-represented in much of the health-related Quality of Life (QoL) research and literature. Disease and its treatment can alter a patient’s ability to fulfil multiple roles and responsibilities such as caring for their own partners, spouses, children and grandchildren or perhaps resuming paid employment. This has a direct bearing not only on an individual’s wellbeing but also for those dependent upon them.

Qualitative studies have demonstrated that roles and responsibilities outside of paid employment, such as being a parent, form significant parts of patients’ lives; cancer has a consequent interaction and impact upon these [8, 9]. There is a direct societal cost also if these areas of concern are not being addressed. One study described how, when compared to patients with early-stage disease, those with metastatic HER2 + breast cancer were less likely to be able to work, even part-time [10].

A workshop held during the Advanced Breast Cancer, 2019 (ABC19) conference involving 92 patient advocates from 27 countries highlighted many commonalties, despite cultural and healthcare delivery differences, in the practical and informational needs of women with advanced/metastatic breast cancer (ABC/MBC) [11]. During this workshop, participants working in small groups discussed issues such as the communication difficulties experienced when receiving their diagnoses and some of the things that healthcare professionals (HCPs), family and friends did or said that either helped or were unhelpful. Significant gaps were identified in patients’ understanding of MBC diagnosis and management, not helped by a dearth of good patient-focussed information. Care and support services were often fragmented and access to clinical trials limited. Importantly the informal caregivers—families and friends—received little guidance as to how best they could support patients.

We wished to determine in more depth, via an online survey conducted within one healthcare delivery system, the experiences of women currently living with MBC in the UK. The specific aim of the project was to utilise any results to inform the content and design of educational resources for patients, their formal and informal health-carers by highlighting areas of concern such as informational gaps, supportive care needs and positive and negative interactions with healthcare providers, family and friends.

## Methods and materials

### Survey development

Data from the ABC19 workshop was used by investigators to draft an initial set of survey questions which five volunteers recruited from MBC support organisations completed. Following their feedback, two further iterations were produced after which a 7-section survey was finalised. There were 29 multiple choice or binary questions and nine that required free text responses. The survey permitted respondents to complete as few or as many of the questions as they wanted.

First the basic socio-educational demographics were recorded, then the wider impact that disease and treatment had exerted upon patients’ lives was assessed using the Patient Roles and Responsibilities Scale (PRRS) [12].

This 29-item measure captures ‘real world’ roles beyond cancer that patients with advanced disease try to maintain such as care-giving obligations for other family members including spouses, children and grandchildren; household tasks; and financial and occupational responsibilities. Respondents indicate on a 5-point scale ranging from ‘not at all’ to ‘very much’ how much of a problem things have been in the past week. One binary response item queries whether the participant has stopped work due to illness. There are 3 subscales—family wellbeing, responsibilities and social life and financial wellbeing together with a standalone scale about jobs and careers comprising 7 items is completed only by those participants currently in paid employment or on sick leave. Negatively worded items were reverse scored so that a higher score corresponds to better quality of life. The maximum possible total score is 60.

Further sections of the survey explored areas such as the clarity of communication with HCPs, who had been present when the diagnosis was discussed; the terms used to describe MBC; explanations regarding treatment and management; and what patients wished they had known about MBC before their diagnosis or still did not understand. The survey probed what other emotional or practical support and resources had been provided and by whom. Patients also described specific aspects of the communication with their HCPs that they perceived as having aided their understanding or ability to cope with the diagnosis and things done or said which did not. Finally, they detailed the various useful practical things that their friends and family could do following a diagnosis of advanced disease, highlighting some of the helpful and less helpful things that people had said or done.

The survey was hosted from October 2021 to February 2022 on the websites of Make2ndsCount, a UK metastatic breast cancer charity based in Edinburgh, Scotland, and SHORE-C. A link was also shared via social media including Twitter. The study had ethical approval from Brighton and Sussex Medical School Research Governance Ethics Committee (RGEC ref: ER/RMLS21/7).

### Statistics

Descriptive statistics (counts and percentages) where appropriate were calculated for the questions that required fixed responses. Some illustrative quotes from free text given to the open-ended questions are presented here which will be reported separately in a thematic analysis paper.

## Results

### Demographics

A total of 143 women with MBC completed the basic demographic questions and the PRRS questionnaire. At least 135 of these women completed all other areas of the survey.

Table 1 shows a summary of respondents’ characteristics as a total and split by first diagnosis (Primary breast cancer or MBC). A third of patients 48/143(34%) had presented with de novo MBC, 95/143 (66%) had developed MBC following a primary breast cancer diagnosis and 54/143(38%) had been living > 2 years with more advanced disease. A majority, 117/143(82%), were partnered or married with 100/143 (70%) citing a spouse or partner as their main support. Many no longer worked, 52/143(36%) had been forced to retire because of their breast cancer but 49/143(34%) were still in paid employment either full or part-time.

**Table 1:** Basic demographics split by 1st diagnosis of primary breast cancer (PBC) or metastatic/advanced breast cancer (MBC)

	1st diagnosis PBC followed by MBC (*n* = 95)	1st diagnosis MBC (*n* = 48)	Combined (*n* = 143)
Age (years)			
(mean; sd)	53.12 (8.78)	48.71 (9.38)	51.64 (9.19)
Min–max range	31–77	28–69	28–77
Ethnicity			
White	86 (91%)	42 (88%)	128 (90%)
Other	9 (9%)	6 (12%)	15 (10%)
How long living with MBC?			
< 1 year	26 (27%)	17 (35%)	43 (30%)
1–2 years	36 (38%)	10 (21%)	46 (32%)
> 2 years	33 (35%)	21 (44%)	54 (38%)
Highest educational level			
No certificates	3 (3%)	-	3 (2%)
O Levels/GCSE	16 (17%)	11 (23%)	27 (19%) 19 (13%)
A Levels	14 (15%)	5 (10%)	
Vocational	15 (16%)	7 (15%)	22 (15%)
University degree	36 (38%)	16 (33%)	52 (37%)
Post grad degree	11 (11%)	9 (19%)	20 (14%)
Marital status			
Married/partner	78 (82%)	39 (81%)	117 (82%)
Not partnered	17 (18%)	9 (19%)	26 (18%)
Main support			
Spouse/partner	66 (70%)	34 (71%)	100 (70%)
Child	8 (8%)	1 (2%)	9 (6%)
Parent	3 (3%)	1 (2%)	4 (3%)
Sibling	5 (5%)	2 (4%)	7 (5%)
Friend	11 (12%)	7 (15%)	18 (13%)
None	2 (2%)	3 (6%)	5 (3%)
Employment Status			
Not in paid employment	12 (13%)	7 (15%)	19 (13%)
Full/part time/self-employed	30 (32%)	19 (40%)	49 (34%)
Retired	20 (21%)	3 (6%)	23 (16%)
Medically retired	15 (15%)	6 (13%)	21 (14%)
Long-term sick leave	18 (19%)	13 (27%)	31 (22%)
Current treatment*			
Surgery	4 (4%)	7(15%)	11 (8%)
Radiotherapy	6 (6%)	6 (13%)	12 (8%)
Chemotherapy (paclitaxel, carboplatin, capecitabine)	38 (40%)	8 (17%)	46 (32%)
Endocrine therapy (anastrazole, exemestane, fulvestrant, letrozole, tamoxifen)	42 (44%)	28 (58%)	70 (49%)
Targeted therapy (abemaciclib, everolimus, palbociclib, ribociclib, trastuzumab)	49 (51%)	34 (71%)	83 (58%)
Immunotherapy (atezolizumbab, pembrolizumab)	14 (15%)	2 (4%)	16 (11%)
Clinical trial	10 (11%)	4 (8%)	14 (10%)
None	6 (6%)	4 (8%)	10 (7%)

^*^Can be on combination of treatments

### Previous and current treatments and management

A total of 82/136(60%) patients were currently having 1st-line treatment and 15/136(11%) had received 4 or more lines of treatment. When asked about palliative/supportive care such as pain control, 42/135(31%) had been offered this of whom 40/42(95%) had accepted it.

Few 19/136(14%) were offered access to MBC clinical trials and of these 14/19(74%) participated. Amongst the 116 women not offered trial participation, 43 (37%) indicated that they would have liked to have been.

### Communication of an MBC diagnosis

The diagnosis of MBC was given usually by an oncologist 63/139(45%) or surgeon 34/139 (24%) and 81/138 (59%) of respondents had met this HCP previously. For 122/139 (88%), the consultation took place in person, 16/139 (12%) had received the news by telephone and one patient via a video call. Only 80/139(58%) had a relative or carer present with them when receiving the diagnosis of advanced disease. Respondents said that HCPs used many different terms to describe breast cancer that had spread outside of the loco-regional area which included MBC, advanced breast cancer (ABC), secondary breast cancer (SBC) and your old problem, your cancer has spread and is now treatable but not curable, terminal/palliative and chronic. The preferred term given by a majority of respondents 75/139(54%) was SBC; they recalled many clinicians using this description but incurable disease or MBC was applied just as often.

Few respondents 16/139(12%) felt that their doctor had delivered prognostic information ‘very clearly’ and 57/139(41%) did not remember it being discussed at all. Less than half 66/139 (48%) recalled anyone checking their understanding about the diagnosis and prognosis although a majority 92/138 (67%) did report having had an opportunity to ask questions during the consultation.

Almost a quarter 32/135 (24%) felt that doctors had considered their lifestyles and/or culture during consultations, although respondents indicated that they had wanted more holistic information including advice about lifestyle, and side-effect management to optimise their quality of life and psychological support.

### Information sources

A majority 118/135 (87%) said that they preferred to receive information about their condition, treatments and management verbally, but had found out the information they needed via a variety of sources, including support groups 89/135 (66%), their own online searches 87/135 (64%), charity websites 83/135 (62%) and through communication with HCPs 81/135 (60%).

Table 2 shows a few examples of the free text answers to more specific questions about information needs and knowledge gaps. In response to the question ‘On reflection is there anything you wished you had known about ABC/MBC before your diagnosis’, 108 women made 191 comments. Several women expressed a general lack of knowledge as to what a diagnosis of MBC actually meant, and what the signs and symptoms were. Others who had first been diagnosed with primary BC appeared to have been unaware of the possibility of breast cancer recurrence or metastasis, and others queried how it had spread and what their prognosis would now be.

**Table 2:** Responses to the free text questions illustrating knowledge gaps

Is there anything you wished you had known about metastatic breast cancer before your diagnosis	‘When diagnosed with primary there was no suggestion that it could return in this way and be incurable. This I would like to have known at primary breast cancer stage’ ‘I wish after my primary it had been discussed & I wish I was told what to look out for & who to contact if I was worried about symptoms’ ‘Just that it existed! I had never heard of it—ALL the focus in the media is on early breast cancer’
Are there things you still do not understand about metastatic breast cancer?	‘How it will progress. The difference between palliative radiotherapy and curative radiotherapy in terms of dosage and expected outcome’ ‘I don’t know how I got it, blood, lymph nodes? I don’t know what order it spread. I do not know which area is the biggest problem. I don’t know if it is hereditary’ ‘Why am I de novo—why haven’t I got a primary?’ ‘How to cope with the emotional effect of my diagnosis and treatment, in particular living with high levels of uncertainty as to what the future might hold’ ‘How targeted therapy works?’ ‘What lifestyle changes really make a difference?’

In response to ‘Are there things you still do not understand about MBC/ABC?’, 61 women offered 132 issues, many of which demonstrated a surprising lack of understanding about fairly basic issues. Some wanted more information from their HCPs, including statistics or expected outcomes from different treatments, extra details about the sites of spread and about their recent test results, including being given the option to look at any imaging scans or mammograms. Issues about clinical trial enrolment were raised again, with many feeling that there was not enough discussion about this and that it was often left to an individual to seek out any trial opportunities.

### Additional support

Just over half of respondents 78/139 (56%) had access to a specialist MBC nurse or key worker and 69/135 (51%) indicated that they had been offered some extra support or help, which was primarily from a specialist nurse 41/69 (59%) or oncologist 23/69 (33%). The additional help was mainly referral to a support group or for psychological services but included assistance with issues such as financial or employment advice, exercise, nutrition and information about complementary therapies. Over two thirds 91/135 (67%) of the respondents had not specifically discussed their emotional needs or the effect that MBC had on their mental health with anyone.

### HCPs’ helpful and less helpful behaviours

The question ‘What things did the healthcare professionals do that helped?’ resulted in 206 comments made by 102 women; ‘What things did not help?’ produced 232 from 94 women. Table 3 illustrates some of these quotes. They revealed that feeling listened to, understood and genuinely cared about was important to patients, as was believing that their individual needs, values and circumstances were acknowledged. Most wished to work collaboratively with their doctor and to be given a genuine role in decision-making, rather than sensing that treatments were being forced upon them. Some respondents shared painful examples of insensitive comments and behaviour or being rushed through the consultation process. Many expressed the importance of continuity and seeing HCPs familiar with their medical history with whom they had built a trusting relationship.

**Table 3:** Illustrative quotes about behaviours that helped and did not help

	Helpful	Not helpful
What things did the healthcare professionals do or say that helped or did not help?	‘Let’s me ask even silly sounding questions’ ‘Respected my knowledge base and treated me like a partner in the treatment team’ ‘My SBCN sat with me for over 2 h when my 1^st^ line (treatment) failed. She really listened; it meant the world to me’ ‘Encouraging about treatments available, gave hope which is what I needed’	‘One of the doctors / locums kept using the term palliative care which upset me greatly as I always associated it with those who are close to end of life’ ‘Being told by a breast care nurse to go home and put my affairs in order!’ ‘Asked me to sign DNR when I went for biopsy’ ‘Oncologist is very matter of fact…. telling a 47 yr old with 2 young children to enjoy life while you can is not helpful’
What did family or friends do or say that helped or did not help?	‘My husband has been amazing just letting me cry’ ‘Big hugs. Lots of unconditional support and love’ ‘My friend came to every appointment and wrote down what was said so that I had a record to read back’ ‘Grandparents had children overnight’	‘my sister and father do not ask how I am, don’t listen very often and don’t remember when I have appointments which hurts’ ‘saying things like you can beat this – no I can’t its terminal, it’s never going away’ ‘being told that I could be hit by a bus tomorrow none of us know how long we have got’? ‘crossing the road so they don’t have to talk to you’

### The supportive roles of friends and family

Respondents were asked to reflect on ‘what things did friends and family do that helped, and what things did not help’. A total of 115 respondents generated 295 quotes (some illustrated in Table 3) which show that the most appreciated and valued practical help and support such as attending appointments with them help with childcare and household chores.

When invited to provide reflections regarding less helpful things friends and family had done, 103 women made 194 comments. These included people ceasing communication, becoming more distant, withdrawn or a fading of some friendships. Some friends and family did not want to say the wrong thing or simply did not know how to respond. Glib comments about staying positive were seen as especially unhelpful.

At the end of the survey, respondents were invited to make suggestions as to ‘What advice would you give to someone recently diagnosed with metastatic breast cancer?’ This produced a large number of statements, 445 from 115 participants; illustrations of these are shown in Table 4. Those diagnosed with MBC at first presentation and those who had lived with advanced breast cancer for longer were more likely to mention the importance of concentrating on the positives and living with hope, with an emphasis on lifestyle and quality of life wherever possible. This included maintaining a sense of identity, making time for enjoyable things in life and liaising with HCPs about how to make these things a priority by arranging treatments around them. Joining an online support group was seen as positive and most importantly ‘Don’t Google’ but rely instead on trusted websites suggested by the clinical team.

**Table 4:** Illustrative quotes about advice to others

What advice would you give to someone recently diagnosed with metastatic breast cancer?	‘Don’t Google! Allow yourself time to process what is happening. And allow family & friends to support you. Get clear information from your oncologist’ ‘Breathe, go easy on yourself, it’s scary but you will have better days through the storm’ ‘Ask about clinical trials and where to go to get access to them’ ‘Don’t panic you’re not going to die tomorrow. Get a plan. Join an online group’ ‘Get over the shock and realise it can be treated although not cured’ ‘Take the time to get your head around your situation. Then let your family and friends in You’ll need them. Put your affairs in place. Then live every day the best you can’

### Day to day roles and responsibilities (PRRS questionnaire)

Almost half of respondents 68/143(48%) had caring responsibilities for children or grandchildren, 11/143(8%) for another adult and 85/143(59%) for pets. Irrespective as to whether their MBC diagnosis followed primary breast cancer or was de novo disease, patients were experiencing a considerable deleterious impact on their everyday roles and responsibilities (PRRS, total score mean 31.48 s.d 11.80).

The PRRS does not have defined thresholds for what constitutes a ‘high’ or ‘low’ score when used at a single time point. The measure has however been used in a number of studies with comparable populations. The women in the LIMBER study reported significantly lower PRRS scores than women with early (stage I/II) breast cancer (mean 48.99, s.d. 12.97), women in other studies with advanced (stage III/IV) breast cancer (mean 37.01, s.d.13.91) and patients with advanced cancers other than breast (mean 39.43, s.d.12.73). Full details are given in Table 5. Over half (58%; 81/140) indicated that their condition had ‘very much’ affected their family wellbeing, and a fifth reported (20%; 29/142) it had greatly affected their responsibilities and social life. Yet, women who managed to continue to work (*n* = 49; 19 of whom were de novo) v those who did not (*n* = 94) had a higher (better QoL) mean total PRRS score (34.26 (s.d. 12.70) v 29.98 (s.d. 11.05) *t*_(138)_ =  − 2.07, *p* = 0.040).

**Table 5:** PRRS total scores across comparable studies

Study	Cancer type	Stage	N*	Mean (S.D.)	*t* (d.f.)	*p*
LIMBER	Breast	IV	140	31.48 (11.80)		
PRAGMATIC + PROACT4	Breast	I/II	122	48.99 (12.97)	− 11.45 (260)	< 0.001
PROACT3 + PROACT4	Breast	III/IV	45	37.07 (13.91)	− 2.64 (183)	0.01
PROACT3 + PROACT4	Mixed other	III/IV	204	39.43 (12.73)	− 5.86 (342)	< 0.001

^*^With valid PRRS total score

## Discussion

Results from the LIMBER study mirrored and expanded many of the findings previously reported by the patient advocates at the ABC19 workshop. [11] A consistent finding in both studies was a lack of awareness that MBC could happen following treatment for primary breast cancer. Breast cancer is characterised by a wide window of relapse that can span months to decades after surgery [13]; thus, patients in the latter category may have considered themselves cured so attribute signs such as back ache to other more benign conditions, for example arthritis or muscle strain rather than to breast cancer again. Not surprisingly, a diagnosis of MBC came as a shock for those who could not recall having been told about the risk of recurrence, or what signs and symptoms to look out for. It is difficult to know if this was a genuine omission by the health care professionals or the consequences of mixed messaging at the end of a treatment consultation, balancing the good news that treatment was effective with cautionary advice.

In the UK, there is a wider move to support people to manage their own follow-up care following hospital-based breast cancer treatments [14]. Although these patients receive verbal and written information at the end of treatment consultation, the literature shows that many leaflets are aimed at people with a higher level of reading ability than commonly present in the general population [15]. Also, those with lower health literacy and/or lack confidence in their ability to recognise symptoms may delay seeking help [16, 17].

LIMBER revealed that for many, the provision of information, help and support was inconsistent. Most participants still had unanswered questions about their treatment and management; it was very noticeable how few said they had received clear information, and that not all HCPs had checked their understanding about the diagnosis/prognosis or gave an opportunity for them to ask questions. For respondents with de novo presentation of advanced disease, the incomprehension and distress when receiving such a diagnosis was profound; this was not helped by the lack of specialist nurses to help support patients with MBC and their families. This reflects the unfortunate lack of resources for these women compared with those receiving an initial diagnosis of a primary breast cancer. A 2010 UK survey found little evidence that women with MBC were routinely allocated a breast care nurse (BCN), and more than half of BCNs reported that care for MBC patients was lacking. [18] As the ratio of patients to nurses increases in the UK, it is unlikely that the situation will resolve swiftly [19]. This is not only a UK problem; an Australian qualitative study of 38 women with MBC identified three themes relating to perspectives of support from their BCNs. The women felt (1) that their supportive care needs are unrecognised, (2) there is confusion over the role and relevance of the BCN to those with MBC and (3) when available the care from the BCN was appreciated, valued and beneficial. [20]

The PRRS data, although collected at only one point in time, revealed the considerable burdens for respondents in terms of their commitments, with a cost to family wellbeing, jobs and careers. Their overall quality of life was considerably lower than either women with EBC or women with other types of advanced cancer. The finding that women who continued to work had better QoL than those who did not could be interpreted in two ways, either employment was ‘normalising’ and a satisfying experience if employers were adaptable or that the ability to work was more a reflection of general physical health status and wellbeing. A recent US survey that explored problems related to employment faced by survivors with MBC reported that 72/133 (54%) continued to work and considered it extremely important to them; the top three cited reasons were (1) financial and/or insurance issues (80%); (2) the need to stay busy (68%); and (3) desire to support themselves and family (64%) [21].

As patients are often reliant on partners, families and friends for emotional, social and practical help, it was disappointing how many respondents felt that their key informal carers were also largely unsupported. We intend to use these survey results in the development of support and educational resources for patients, their families and health care professionals. The first of which is a short film entitled ‘They just don’t know what to say or do’ aimed at relatives and friends of patients with MBC. A thematic analysis of the rich and sometimes moving free text comments is being prepared and will form the basis of further educational materials.

## Conclusion

Navigating the complexities and ramifications of a diagnosis of MBC is difficult for all concerned. Results from the LIMBER survey reported here gave an opportunity to examine patients’ perspectives from within one healthcare system and to analyse more deeply through quantitative and qualitative methods those areas of concern which might be modifiable. MBC treatments are constantly improving, providing more patients with a genuine prospect of living longer so we also need to enhance the quality of that extra survival through the provision of better information and support services.

## Data Availability

The datasets generated during and/or analysed during the current study are available from the corresponding author on reasonable request.

## Declarations

### Disclaimer

The Charity had no role in the collection, analysis or interpretation of the data or in the writing of the manuscript.
